# The Fibrotic Phenotype of Human Precision-Cut Lung Slices Is Maintained after Cryopreservation

**DOI:** 10.3390/toxics12090637

**Published:** 2024-08-30

**Authors:** Méry Marimoutou, Vivek Patel, Jae Hun Kim, Niccole Schaible, Jose Alvarez, Joseph Hughes, McKenzie Obermok, Carlos Iván Rodríguez, Thomas Kallarakal, Béla Suki, Khalid Amin, Ramaswamy Krishnan, Holger Peter Behrsing

**Affiliations:** 1 Institute for In Vitro Sciences, Inc., Gaithersburg, MD 20878, USA; 2 Mechanobiologix, LLC, Newton, MA 02464, USA; 3 Department of Biomedical Engineering, Boston University, Boston, MA 02215, USA; 4 Center for Vascular Biology Research, Department of Emergency Medicine, Beth Israel Deaconess Medical Center and Harvard Medical School, Boston, MA 02215, USA; 5 Department of Laboratory Medicine and Pathology, University of Minnesota, Minneapolis, MN 55455, USA

**Keywords:** fibrosis, cryopreservation, PCLS, IPF, human, stiffness, non-animal testing, ex vivo, in vitro lung models

## Abstract

Human precision-cut lung slices (hPCLS) prepared from fibrotic lungs recapitulate the pathophysiological hallmarks of fibrosis. These hallmark features can also be induced by treating non-fibrotic hPCLS with a fibrotic cocktail (FC). As a result, the fibrotic and fibrosis-induced hPCLS are rapidly emerging as preferred models for disease modeling and drug discovery. However, current hPCLS models are limited by tissue viability in culture, as they are usually only viable for one week after harvesting. Here, we demonstrate that the fibrotic hPCLS can be cryopreserved, stored for months, and then thawed on demand without loss of hPCLS viability or protein content for 14 days post-thawing. Cryopreservation also preserves the pro-fibrotic potential of non-fibrotic hPCLS. Specifically, when we treated the thawed non-fibrotic hPCLS with an FC, we observed significant pro-fibrotic cytokine secretion and elevated tissue stiffness. These pro-fibrotic changes were inhibited by the small-molecule tyrosine kinase inhibitor, Nintedanib. Taken together, our work indicates that a feasible solution to prolong the pre-clinical utility of fibrotic and fibrosis-induced hPCLS is cryopreservation. We anticipate that cryopreserved hPCLS will serve as an advantageous predictive model for the evaluation of pro-fibrotic pathways during acute and chronic toxicity testing.

## 1. Introduction

Fibrotic Interstitial Lung Disease (ILD) encompasses a diverse group of lung disorders characterized by parenchymal stiffening and scarring [1,2]. Affected individuals experience progressively worsening breathing difficulties that can eventually lead to respiratory failure. The most common fibrotic ILD subtype, Idiopathic Pulmonary Fibrosis (IPF) [3], affects nearly 3 million people worldwide, with an estimated prevalence as high as 400 cases per 100,000 people in patients over the age of 65 [4,5]. IPF patients have a very poor prognosis, with an average life expectancy of only 3 to 5 years following initial diagnosis [6]. Unfortunately, IPF’s prevalence is on the rise, with approximately 50,000 new cases diagnosed annually in the United States alone [7]. While current treatments can temporarily improve IPF symptoms or slow the rate of lung function decline [8], IPF remains incurable.

To find new IPF cures, academic and pharmaceutical leaders have emphasized the need to develop new pre-clinical models that closely resemble the human lung [9,10,11,12]. Ideally, these models must incorporate composite endpoints that include “feels, functions, and survives” outcomes [13]. To this end, viable preparation of thin slices of human lungs, referred to as human precision-cut lung slices (hPCLS) [14], are advantageous. Compared to 2D cell cultures, 3D organoids, reconstructed tissues grown at the air–liquid interface, and lung microphysiological systems [15,16,17,18], hPCLS have a unique ability to retain the native multicellularity, 3D architectural arrangement, and extracellular matrix (ECM) composition of the human lung, including those of diseased tissues. hPCLS can be prepared from IPF/ILD lungs [19] or early fibrotic changes can be induced in hPCLS derived from patients without IPF/ILD [20]. The fibrotic and fibrosis-induced hPCLS recapitulate excessive collagen deposition, alveolar epithelial reprogramming, fibroblast-to-myofibroblast differentiation [21], and spatiotemporal heterogeneity amongst localized fibrotic lesions, with areas of normal lung architecture interspersed with regions of stiff fibrotic foci comprising fibroblasts and α smooth muscle actin-positive myofibroblasts [22]. Notably, in hPCLS, it is possible to directly evaluate physiological outcomes relevant to patients’ wellbeing, including parenchymal stiffness [23], stretch [24,25,26], airway constriction [14,27,28,29], and pro-fibrotic secretion [30]. Additional practical advantages of hPCLS are their ease of pharmacological manipulation; their ability to non-invasively measure longitudinal changes in fibrotic progression, including collagen synthesis and accumulation, cytokine production, and morphological changes [19,20,30,31,32,33,34,35]; and their amenability to genetic manipulations [36], RNA isolation [37], histology [20], and mechanotransduction assays [26]. Beyond IPF, hPCLS are widely enabling studies of airway inflammation [38], viral infection [39], bronchoconstriction [28], early-stage COPD [26], host–pathogen interactions [40], chemical toxicity [41], and drug safety, efficacy, and donor-to-donor variability [42]. Taken together, hPCLS appear ideally suited to bridge the translational gap between pre-clinical findings and fibrotic outcomes in humans.

The utilization of hPCLS was traditionally limited to a few days from tissue harvest [14]. Improvements in lung procurement, slice preparation, and culture media conditions have since enhanced hPCLS’ viability, allowing them to be stored for months [43,44,45,46]. The incorporation of cryopreservation [27,38,46] has enhanced hPCLS use even further, to almost a year [46]. Using cryopreservation, thousands of hPCLS created from a procured human lung can be frozen and stored long-term, to be thawed on demand for later use. We and others have demonstrated that freezing–thawing has minimal impact on overall tissue integrity, biomass, cell viability, mitochondrial integrity, glutathione activity, airway caliber responses to contractile and relaxant agonists, toxicological responses, cytokine secretion to inflammatory stimuli, and immune cell functions, including phagocytosis and lymphocyte proliferation [27,38,41,46]. Logistically, because tissue procurement becomes uncoupled from tissue testing, utilization of precious samples is maximized. Cryopreservation also obviates difficulties with lung procurement delays. Finally, cryopreservation enables tissue banking, especially of diseased tissues. These advantages have fostered expanded hPCLS use in studies of bronchodilator drug discovery, predictive toxicology, and COPD pathophysiology [23,41,45]. However, cryopreservation has never been demonstrated to maintain hPCLS’ fibrotic phenotype.

Our key findings are as follows: (1) Cryopreservation preserves the viability, protein content and immune responsiveness of the fibrotic hPCLS, and, (2) cryopreservation preserves the pro-fibrotic potential of non-fibrotic hPCLS. These results are significant because cryopreservation enables hPCLS banking for future use in fibrotic and fibrotic-induction studies.

## 2. Materials and Methods

### 2.1. Human Lung Procurement

Non-transplantable de-identified lungs were obtained from consenting donors (or next of kin) through accredited procurement agencies, including the International Institute for the Advancement of Medicine (IIAM; Edison, NJ, USA), Novabiosis, Inc. (Durham, NC, USA), or National Disease Research Interchange (Philadelphia, PA, USA). The donor demographics and hPCLS information for the hPCLS fibrosis study are provided in Table 1.

Microphotographs from an additional normal donor (N6; 62 years old, Caucasian, 183 cm in height, 106.5 kg, male; not part pf the fibrosis study) were taken to compare H&E-stained sections and demonstrate the lack of overt differences between never-frozen and frozen–thawed hPCLS.

### 2.2. hPCLS Preparation

Lungs were inflated using a balloon catheter with prewarmed (38–42 °C) agarose (Molecular Biology Grade, bioWORLD, Dublin, OH, USA, cat. no. 40100104-3) solution (0.8% in HBSS, Bioworld, Dublin, OH, USA), and then cooled (2–8 °C) for approximately 45 min to facilitate the agarose solution’s transition to gel form. From the solid-gelled inflated lungs, we cut 1–1.5 cm thick sections using a scalpel and placed the sections in cold slicing buffer [46]. The sections were cut further using a MD2300 or MD5000 low-speed coring press (Alabama Research and Development, Munford, AL, USA) to generate cylindrical cores (~8 mm diameter). Finally, the cores were thinly sliced (400–500 µm thickness) using the Krumdieck MD4000 tissue slicer (Alabama Research and Development) according to the manufacturers’ protocols. The hPCLS batches were collected and stored (<24 h) in cold slicing buffer until they underwent culture or cryopreservation.

### 2.3. hPCLS Cryopreservation and Thawing

Up to 6 hPCLS samples were carefully placed (using a sterile cotton swab or fine-tip forceps) into a cryovial containing approximately 1.5 mL cryopreservation buffer (CB) (proprietary; made at the Institute for In Vitro Sciences (IIVS), Gaithersburg, MD) and then frozen at ≤−60 °C at an approximate rate of −0.4 to −1.5 °C/minute for at least 4 and up to 72 h. The cryovials were then transferred to the vapor phase of a liquid nitrogen tank for long-term storage [46]. The length of time of frozen storage prior to use in experimentation was variable for each donor (mean = 205 ± 162 days for all batches thawed). To prepare the hPCLS for use, the cryovials were removed from storage, rapidly thawed, and rinsed twice in 12 mL prewarmed (37 °C) acclimation medium, and acclimated in multiwell plates until use.

### 2.4. hPCLS Acclimation

Following slice creation or directly after thawing, each hPCLS was submerged in 1 mL acclimation medium (AM) for 1–3 days. The acclimation media was composed of Dulbecco’s Modified Eagle Medium/Nutrient Mixture F-12 (DMEM/F-12, Rmbio, Missoula, MT, USA, Cat. no. DME-BBZ-01L-003) with 1% GlutAmax™ (Gibco, Waltham, MA, USA, Cat. no. 35050-061), 1% Insulin-Transferrin-Selenium (ITS-G; Gibco, Cat. no. 41400045), and 0.2% Primocin^®^ (InvivoGen, Cat. no. PML-44-03, San Diego, CA, USA) supplemented with 1% Antibiotics-Antimycotics (Sigma-Aldrich, St Louis, MO, USA, Cat. no. A5955100ML), 2 µM Hydrocortisone (Sigma-Aldrich, Cat. no. H0396-100MG), and 10 µg/mL Ascorbic acid (Sigma-Aldrich, Cat. no. 49752-100ML).

### 2.5. hPCLS Culture 

After the acclimation period, the hPCLS was placed in culture medium (CM) at standard culture conditions (SCC; i.e., 37 ± 1 °C, 5% CO_2_, and 90% relative humidity) for up to 14 days. The culture medium was composed of DMEM F-12 (Rmbio, Cat. no. DME-BBZ-01L-003) with 1% GlutAmax™ (Gibco, Waltham, MA, Cat. no. 35050-061), 1% Insulin–Transferrin–Selenium (ITS-G; Gibco, Cat. no. 41400045), and 0.2% Primocin^®^ (Invivogen, Cat. no. PML-44-03). 

### 2.6. hPCLS Treatments

(1) Fibrotic cocktail (FC): Stock solutions of Tumor Necrosis Factor-α (TNF-α) (R&D Systems, Minneapolis, MN, USA, Cat. no. 210-TA/CF) (100 µg/mL in PBS), 1-Oleoyl Lysophosphatidic Acid (sodium salt; LPA) (Cayman Chemicals, Ann Arbor, MI, USA, Cat. no. 62215) (5 mg/mL in PBS), Recombinant Transforming Growth Factor-β (TGF-β) (R&D Systems, Minneapolis, MN, USA, Cat. no. 7754-BH) (100 µg/mL in 4 mM HCl), and Platelet-derived Growth Factor-AB (PDGF-AB) (R&D Systems, Cat. no. 222-AB-010) (100 µg/mL in 4 mM HCl) were solubilized in CM to prepare the FC at final doses of TGF-β (5 ng/mL), PDGF-AB (10 ng/mL), TNF-α (10 ng/mL), and LPA (5 µM). (2) Control cocktail (CC): Appropriate volumes of vehicle (i.e., HCl (4 mM) and PBS) corresponding to the amount of the diluents added to FC were added to the CM. (3) We solubilized Nintedanib (Nin): 5 mg (Sigma, Cat. no. SML2848-5MG) in DMSO (Sigma, Cat. no. D2650-100ML) to prepare 10 mM concentration stock. The stock was diluted further in CC or FC culture media to achieve a final concentration of 1 µM. All three groups were treated at D0 and then treated again every 2 days for up to 4 days.

### 2.7. WST-8 Viability Assay

At D7 and D14, we carefully removed hPCLS from their culture vessel and placed them into wells of a 24-well plate containing 500 μL CM mixed with WST-8 substrate (10:1 ratio, respectively). After 2 h of incubation in standard incubator conditions, we measured WST-8 conversion (as a function of viability) by removing 100 μL of incubation medium from each well, transferring it to a 96-well plate, and measuring for absorbance at 450 nm on a plate reader. Background readings (CM mixed with WST-8 substrate) were subtracted to obtain final OD450 scores.

### 2.8. Cytokine/Biomarker Analysis

Fibronectin, pro-collagen Iα1, MMP-3, MMP-7, TIMP-1, IL-6, and TNFα secretion were assessed using Luminex assay kit (R&D Systems, [cat # LXSAHM]). The samples were prepared as instructed in the kit manual and run on Luminex MAGPIX system (Luminex Corporation, Austin, TX, USA). We analyzed the data using the Luminex xPONENT^®^ Version 4.3 software.

### 2.9. Total Protein Content

Total protein content (as an indicator of biomass) was assessed using the BCA protein assay kit (ThermoFisher Scientific, Waltham, MA, USA, [cat. no. 23227]). Briefly, the hPCLS was lysed and 25 μL of lysate (and standards) was added in duplicate to a 96-well plate. An additional 200 μL of the BCA working reagent was added to each well, and the plate was then placed in an orbital shaker at 800 rpm for 30 s at room temperature. The plate was then incubated at SCC for 30 min and, finally, analyzed for absorbance at 562 nm using the VersaMax microplate reader.

### 2.10. hPCLS Stiffness

We have recently developed a biomechanical device for measuring hPCLS stiffness called the MechanoWell^®^ [23]. This device comprises (1) an elastic composite substrate consisting of an elastic silicon membrane and a coating layer of soft polydimethylsiloxane (PDMS) gel (NuSil 8100^®^ Silicone Technologies, Carpinteria, CA, USA), (2) fluorescent beads embedded near the top surface of the composite, and (3) a setup which sits on an inverted microscope and applies equibiaxial stretch (10% magnitude) to the composite and an adhered sample. After an hPCLS was adhered to the composite substrate and equibiaxially stretched, the distribution of displacements and strains underneath the hPCLS was mapped across a ~2 mm × 2 mm field of view by tracking the fluorescent beads. From the strains, measured without and then with the hPCLS, and with additional measurement of hPCLS thickness, we used a look-up table (Figure 1C in [23]) to estimate the hPCLS stiffness. 

### 2.11. Statistics

Unless otherwise indicated, statistical comparisons of cytokine secretion were performed using one-way ANOVA and Sidak’s multiple comparisons test. Differences were considered significant at *p* < 0.05. Data are reported as the mean and standard deviation or standard error of the mean, as well as distribution. Distributions and lognormal fits were performed in MATLAB R2021a (MathWorks, MA, USA). A Bayesian *t* test was carried out in JASP 0.16 (University of Amsterdam). Posterior distributions were calculated in MATLAB using a custom code that assumed unknown mean and variance in the form of a two-parameter gamma-normal distribution and a normal distribution for the likelihood function. To avoid bias, nearly uniform prior distributions were used in the calculations. Finally, for each biological outcome, we report both the averaged data and a donor-to-donor breakdown.

## 3. Results

### 3.1. Freezing–Thawing the Fibrotic hPCLS Does Not Affect Viability or Protein Content

**Figure 1:**
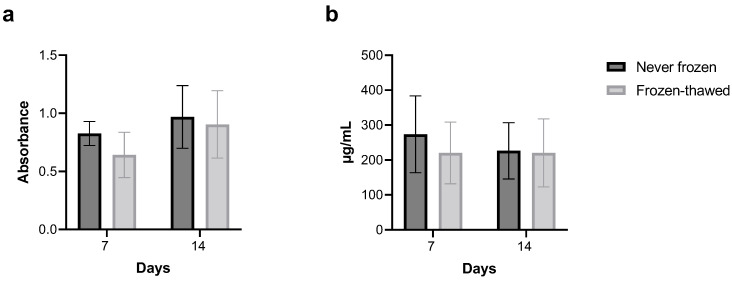
Freezing-thawing the fibrotic human PCLSs (hPCLS) does not affect their viability or protein content. (**a**) WST-8 assay shows no significant difference between never-frozen and frozen–thawed groups kept in culture for up to 14 days. Data are reported as the absorbance of WST-8 formazan at 450 nm, a value that is proportional to the number of viable cells in the medium. (**b**) BCA assay revealed no differences in protein concentration between the never-frozen and frozen–thawed groups kept in culture for up to 14 days. Each bar is an average and standard deviation over five fibrotic donors, with n = 6 hPCLS per donor for the frozen–thawed group and n = 3 hPCLS per donor for the never-frozen group. One-way ANOVA and Sidak’s test were used. See Supplementary Figure S2 for a donor-to-donor breakdown.

The WST-8 assay shows that viability was comparable between the never-frozen and frozen–thawed groups (Figure 1a). Additional measurements of hPCLS protein content using the BCA assay revealed no differences between the never-frozen and frozen–thawed groups (Figure 1b). These data are consistent with our previous findings in cryopreserved non-fibrotic hPCLS [46] and as shown in Supplementary Figure S1.

### 3.2. Freezing–Thawing the Fibrotic hPCLS Largely Preserves Pro-Fibrotic Cytokine and MMP Secretion

**Figure 2:**
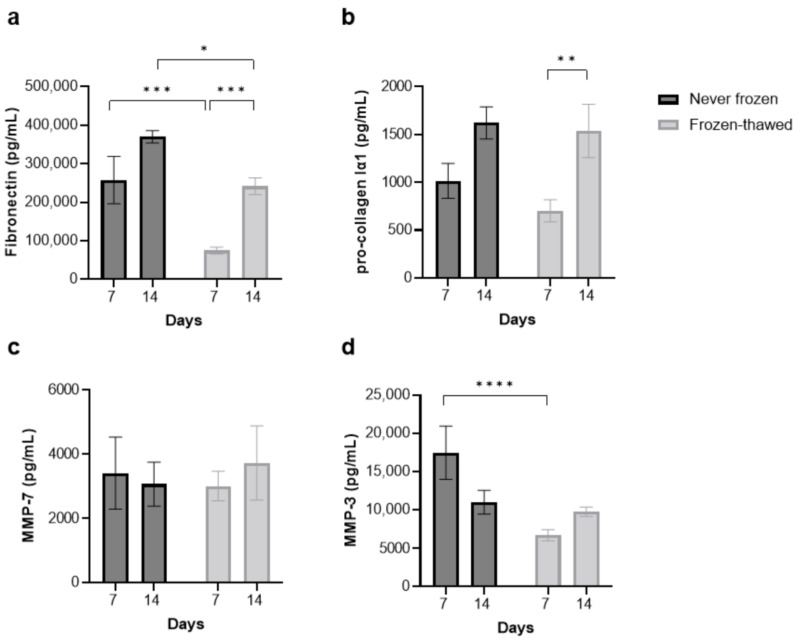
Freezing–thawing the fibrotic hPCLS largely preserves pro-fibrotic cytokine secretion. (**a**–**d**) MAGPIX^®^ analysis of cytokine production revealed that with the exception of fibronectin and MMP-3, each of the pro-fibrotic cytokines were secreted comparably between the never-frozen and frozen–thawed fibrotic hPCLS at day 7. All cytokines were secreted comparably on day 14. Each bar represents the mean and standard error of mean over five fibrotic donors, with n = 6 hPCLS per donor for the frozen–thawed group and n = 3 hPCLS per donor for the never frozen group. One-way ANOVA and Sidak’s test were used. *: *p* < 0.05, **: *p* < 0.01, ***: *p* < 0.001, ****: *p* < 0.0001. See Supplementary Figure S3 for a donor-to-donor breakdown.

MAGPIX^®^ analysis revealed that except for fibronectin and MMP-3, each of the pro-fibrotic cytokines were secreted comparably between the never-frozen and frozen–thawed fibrotic hPCLS at D7 and D14 (Figure 2). In the case of fibronectin and MMP-3, while a significant difference was observed at D7, it was restored to comparable levels at D14. 

### 3.3. Freezing–Thawing the Fibrotic hPCLS Largely Preserves Pro-Inflammatory Cytokine Secretion

**Figure 3:**
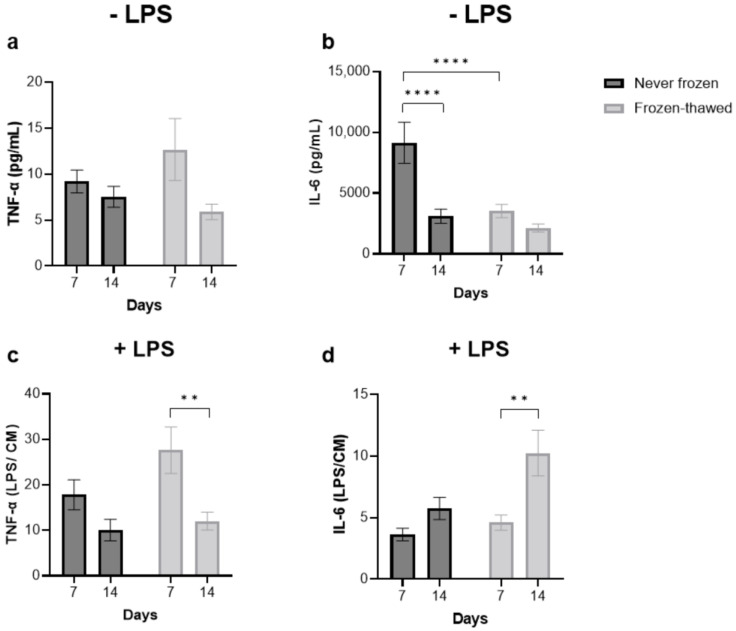
Freezing–thawing the fibrotic hPCLS largely preserves pro-inflammatory cytokine secretion. (**a**,**b**) MAGPIX^®^ analysis of endogenous cytokine production revealed that compared to the never-frozen hPCLS, the frozen–thawed fibrotic hPCLS secreted similar levels of TNF-α (**a**) but significantly less IL-6 (**b**) at D7. However, these differences were absent at D14. (**c**,**d**) With subsequent LPS treatment, the average fold change (relative to pre-treatment) was comparable between the never-frozen and frozen–thawed fibrotic hPCLS at both D7 and D14. Each bar represents the mean and standard error of mean over five fibrotic donors with n = 6 hPCLS per donor for the frozen–thawed group and n = 3 hPCLS per donor for the never-frozen group. One-way ANOVA and Sidak’s test were used. **: *p* < 0.01, ****: *p* < 0.0001. See Supplementary Figure S4 for a donor-to-donor breakdown.

MAGPIX^®^ analysis of endogenous cytokine production revealed that compared to the never-frozen hPCLS, the frozen–thawed hPCLS secreted similar levels of TNF-α (Figure 3a) but significantly less IL-6 at D7 (Figure 3b). However, these differences were absent at D14. With subsequent LPS treatment (chosen due to its ability to produce robust immune responses, including exacerbations in IPF models [47]), both the never-frozen and frozen–thawed fibrotic hPCLS demonstrated similar fold enhancement of both TNF-α (Figure 3c) and IL-6 (Figure 3d). 

### 3.4. FC Treatment of the Frozen–Thawed Non-Fibrotic hPCLS Induces Pro-Fibrotic, Pro-Inflammatory, and MMP Secretion

**Figure 4:**
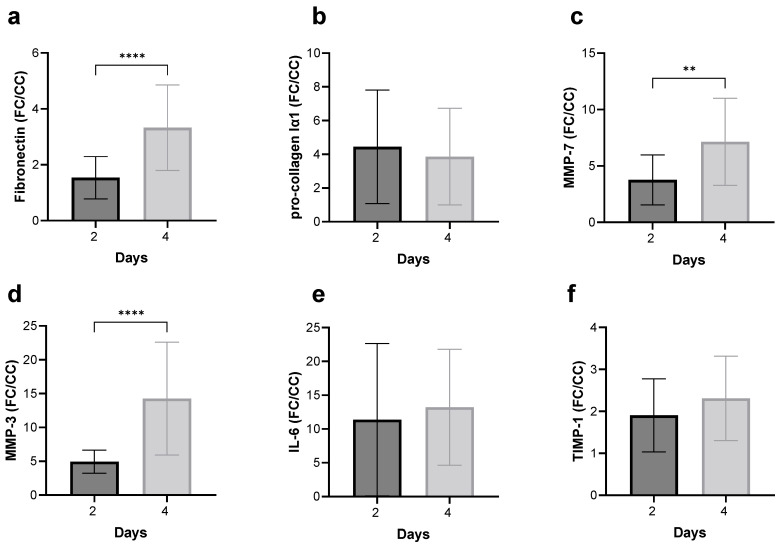
Fibrotic cocktail (FC) treatment of the frozen–thawed healthy hPCLS induces pro-fibrotic and pro-inflammatory secretion. (**a**–**f**) MAGPIX^®^ analysis of pro-fibrotic and pro-inflammatory secretion revealed that compared to the control cocktail (CC)-treated hPCLS, FC-treated hPCLS secreted at least 2-fold and up to 15-fold greater levels of cytokines. In the case of fibronectin, MMP-3, and MMP-7, the amount of secretion was also time-dependent. Each bar represents the mean and standard deviation of fold changes (i.e., FC-induced cytokine secretion normalized to CC-induced cytokine secretion, matched for treatment day and donor) over four normal donors, with n = 6 hPCLS per donor. **: *p* < 0.01, ****: *p* < 0.001. See Supplementary Figure S5 for a donor-to-donor breakdown.

MAGPIX^®^ analysis of pro-fibrotic and inflammatory secretion revealed that compared to the CC-treated frozen–thawed non-fibrotic hPCLS, the FC-treated frozen–thawed non-fibrotic hPCLS secreted at least 2-fold and up to 15-fold greater levels of pro-fibrotic and pro-inflammatory cytokines (Figure 4a–f). In the case of fibronectin (Figure 4a), MMP-7 (Figure 4c), and MMP-3 (Figure 4d), the amount of secretion was greater at day 4 compared to day 2. 

### 3.5. FC Treatment of the Frozen–Thawed Non-Fibrotic hPCLS Induces hPCLS Stiffening

**Figure 5:**
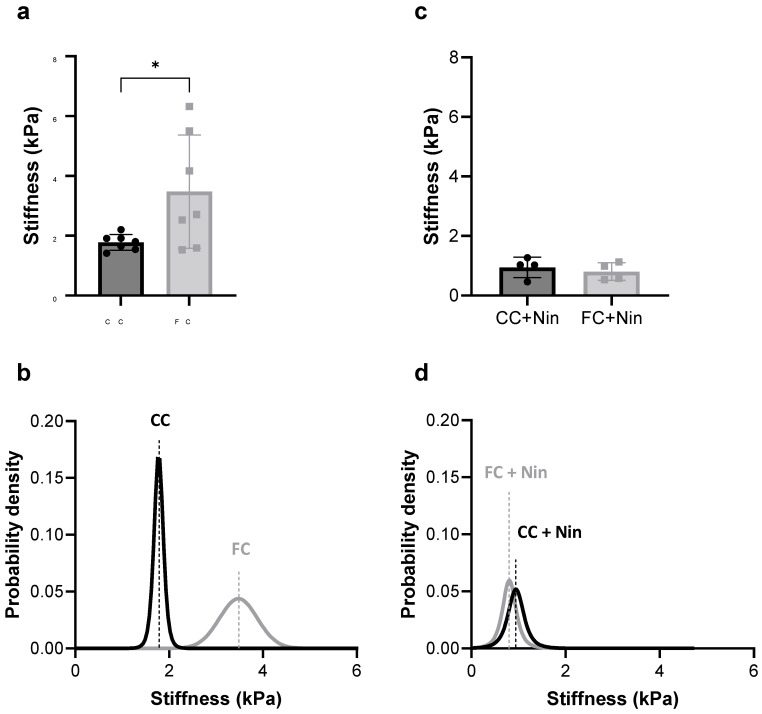
FC treatment of the frozen–thawed healthy hPCLS induces tissue stiffening. Such stiffening can be inhibited by co-treatment with Nintedanib. Biomechanical measurements of hPCLS stiffness at D4 revealed that (**a**) compared to the CC-treated frozen–thawed healthy hPCLS, the FC-treated frozen–thawed healthy hPCLS were significantly stiffer. n = 7 hPCLS per group, donor N3. (**b**) Bayesian estimation of the posterior distribution of stiffness of CC- and FC-treated hPCLS. The distributions do not overlap (one-tailed Bayesian factor: 12.54). (**c**) FC-induced stiffening was reduced by Nintedanib (Nin) co-treatment. (**d**) Bayesian estimation of the posterior distribution of stiffness of CC + Nin- and FC + Nin-treated hPCLS. The circles (CC) and squares (FC) on the respective bar graphs (**a**,**c**) represent individual values that contributed to the mean and variation depicted by each bar. No significant difference was observed between the CC + Nin and the FC + Nin groups. n = 4 hPCLS per group, donor N5. *: *p* < 0.05.

Biomechanical measurements of hPCLS stiffness revealed that compared to the CC-treated frozen–thawed non-fibrotic hPCLS, the FC-treated frozen–thawed non-fibrotic hPCLS were, on average, 50% stiffer (Figure 5a). To further compare the stiffness of the CC-treated and FC-treated hPCLS, we performed a Bayesian analysis of the mean stiffness (Figure 5b). The posterior distributions in Figure 5b show little overlap between them with a one-sided Bayesian factor of 12.6. It is thus highly unlikely that the null hypothesis, that the CC-treated hPCLS would have the same mean as the FC-treated hPCLS, is true.

### 3.6. Nintedanib Co-Treatment Inhibits FC-Induced Stiffening and Pro-Fibrotic Secretion

**Figure 6:**
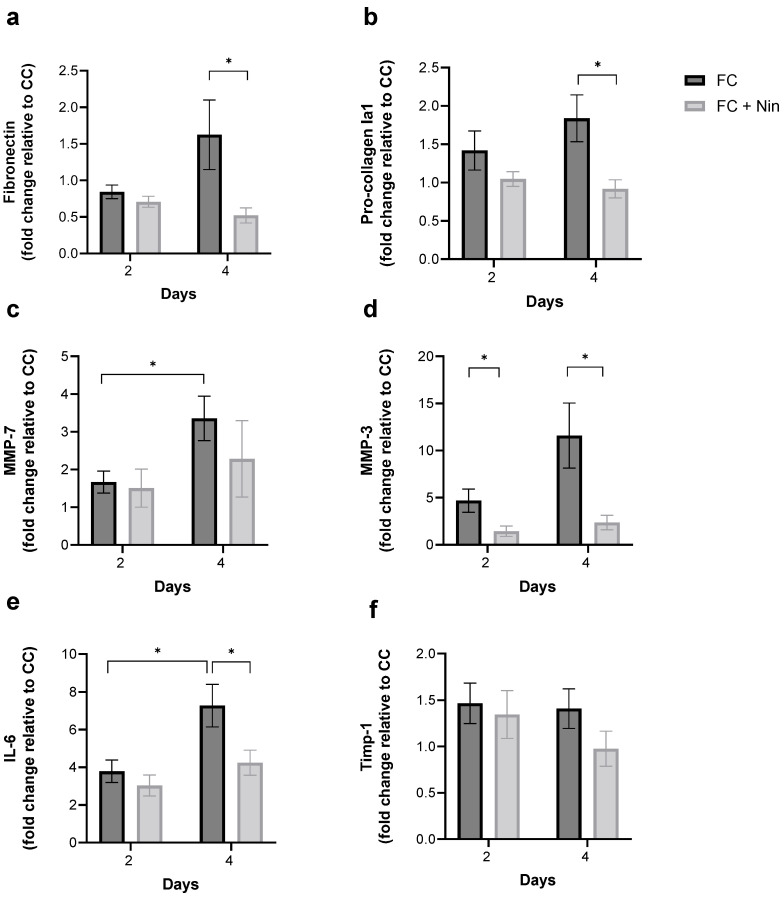
Nintedanib reduces FC-induced pro-fibrotic secretion in the frozen–thawed healthy hPCLS. (**a**–**f**) MAGPIX^®^ analysis of cytokine production revealed that Nintedanib reduces FC-cocktail induced pro-fibrotic secretion with significant reduction in the cases of fibronectin (D4), pro-collagen Ia1 (D4), MMP-3 (D2 and D4), IL-6 (D4). Data are reported as the fold change relative to donor-matched CC-treated hPCLS. Each bar represents the mean and standard error of mean over two normal donors (N4 and N5), with n = 6 hPCLS per donor. Student’s *t* test was used (*: *p* < 0.05). See Supplementary Figure S6 for a donor-to-donor breakdown.

Biomechanical measurements of hPCLS stiffness revealed that Nintedanib (chosen because of its known impact on collagen turnover in fibrotic slice tissue [30]) co-treatment inhibited FC-induced stiffening of the frozen–thawed non-fibrotic hPCLS, to an extent comparable to the CC + Nintedanib-treated group (Figure 5c). Additional Bayesian analysis of the mean stiffness (Figure 5d) revealed strongly overlapping posterior distributions with a one-sided Bayesian factor of 1.7. It is thus highly likely that the FC + Nintedanib-treated hPCLS had the same mean as the CC + Nintedanib-treated hPCLS. Moreover, measurements of pro-fibrotic and inflammatory secretion using MAGPIX^®^ analysis revealed that Nintedanib co-treatment inhibited FC-induced cytokine enhancements, with significant reductions in the case of fibronectin, pro-Collagen Iα1, MMP-3, and IL-6 at D4 (Figure 6).

### 3.7. Inter-Sample Variability of the Data Following FC Treatment

**Figure 7:**
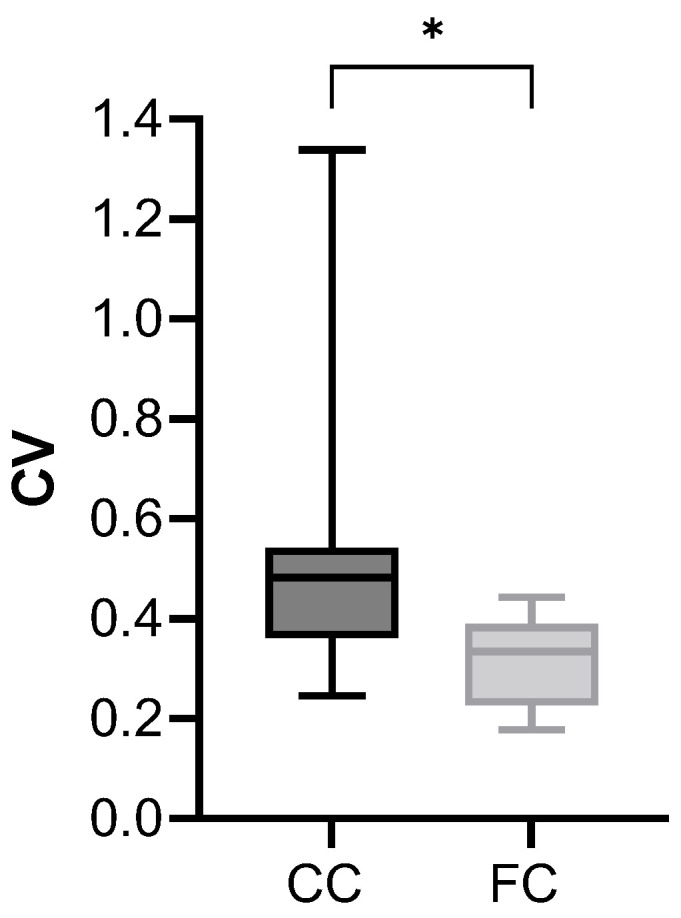
Coefficient of variation of the biomarkers following 4 days of CC or FC treatment. The coefficient of variation, defined as SD over mean, for each biomarker were computed at days 2 and 4 and the effects of FC compared to CC were evaluated using a 2-way ANOVA. There was no difference between D2 and D4 data and hence these groups were combined to evaluate the effects of FC via a Mann–Whitney rank sum test: * *p* < 0.007.

The coefficient of variation (CV = SD/mean) of stiffness increased from 14.8% following CC to 54.4% after FC treatments (F test, *p* = 0.006). This is consistent with the expected increase in structural variability as the disease progresses. Unexpectedly, however, the CV of all biomarkers decreased substantially and statistically significantly (Figure 7) from 48.3% after CC to 33.4% after FC treatment (*p* = 0.007). 

## 4. Discussion

In this study, we have demonstrated (1) that freezing and thawing of the fibrotic hPCLS preserves viability, protein content, and immune responsiveness and (2) the utility of cryopreservation toward fibrotic disease modeling. These findings are of practical importance because cryopreservation permits on-demand and ready-to-use donor-specific hPCLS for fibrosis studies. These studies comprise not only the evaluation of therapeutic efficacy but also the potential adverse pro-fibrotic effects of nanoparticles, biologicals, and environmentally prevalent materials. Thus, our approach to fibrotic modeling using the frozen–thawed hPCLS will inform epidemiological risk assessment as well as contribute to our understanding of IPF disease etiology. To this end, it is well known that multiple factors (e.g., environmental and occupational exposures, bacterial and viral infections, drugs, radiation, and genetic predisposition) have been implicated in disease pathogenesis [48,49,50], and after individuals have developed this debilitating disease, a fibrosis model (i.e., the fibrotic hPCLS) that helps us understand the increased risks to this predisposed population is invaluable. Further, the ability to induce the fibrotic phenotype in frozen–thawed normal hPCLS supports the need for a second model that can allow the evaluation of pro-fibrotic induction. While further work is required to explore the latter, our studies with the FC presented here indicate that the required cellular mechanisms are present (and retained after cryopreservation and thawing) and that 2+ week cultures are possible. Unpublished data generated by one of the authors (H. Behrsing) with fresh wildtype rat lung slices indicate that multi-well exposure to bleomycin or carmustine (a chemotherapeutic with known pro-fibrotic effects in patients) can generate significant collagen deposition—a hallmark of the fibrotic phenotype. 

To measure cryopreservation and drug intervention effects in fibrotic and fibrosis-induced hPCLS, we studied common ECM turnover markers in the hPCLS, including pro-collagen Iα1, fibronectin, MMPs [30], and inflammatory markers, including IL-6 and TNFα [51]. Except for fibronectin (Figure 2a) and MMP-3 (Figure 2d) at D7, each of these markers were comparable between the never-frozen and frozen–thawed fibrotic hPCLS at both D7 and D14. In the case of fibronectin and MMP-3 at D7, reduced expression in the frozen–thawed fibrotic hPCLS might be related to known cell metabolic reduction in the hPCLS immediately after thawing [38]. Regardless of these effects, at both D7 and D14, the never-frozen and frozen–thawed fibrotic hPCLS secreted comparable fold amounts of TNFα (Figure 3c) and IL-6 (Figure 3d) with LPS stimulation. Finally, the pro-fibrotic and pro-inflammatory effects were qualitatively consistent across multiple donors (Supplementary Figures S1–S3).

The effects of FC were to increase the levels of cytokines and MMPs (Figure 4) as well as to increase the stiffness (Figure 5). Interestingly, the CV of stiffness increased, but the CV for all biomarkers decreased significantly after FC treatment. 

The disparity between the stiffness and biomarker CVs may be explained as follows. During CC treatment, cells produce and secrete various molecules, including those that we measured. During FC challenge, fibrotic pathways are reinforced and cells start to uniformly secrete more of the corresponding molecules; hence, the CV of this process is reduced. The secretion of inflammation-enhancing cytokines and MMPs as well as various collagens will act in a positive feedback loop to locally further stiffen an already stiff region, leading to both an increased stiffness, amplified structural heterogeneity [52], and cumulatively, an increased CV of stiffness.

While biomarkers are useful surrogate measurements of fibrotic induction, they might not reliably reflect holistic functional changes in tissue remodeling. Thus, we validated fibrotic induction through direct measurements of tissue stiffness. We found that the fibrosis-induced hPCLS at D4 was ~50% stiffer than the non-fibrotic hPCLS (Figure 5a,b). However, not all slices stiffened to the same extent, despite corresponding enhancement of pro-fibrotic secretion (Figure 4, Supplementary Figure S5). This difference likely reflects the slice-to-slice heterogeneity in ECM expression and structure, especially cross-linking, and reiterates the importance of quantifying physiological outcomes. In this connection, a key advantage of stiffness measurements over collagen synthesis and accumulation, myofibroblast expression, and cell biological changes [19,20,30,31,32,33,34,35] is that stiffness changes are not tethered to specific cell types, structural changes, or molecular pathways. Thus, the entire vast diversity of molecular targets in the cell and/or ECM becomes accessible to therapeutic screening via stiffness measurements. Moreover, because lung softening is the endpoint of therapeutic intent in fibrosis, direct stiffness measurements will reduce the possibility of false-positive and false-negative leads. Finally, our approach to stiffness measurements is label-free, non-invasive, rapid (<3 min per measurement), and readily integrated with traditional fibrotic drug-discovery endpoints. For all these reasons, we anticipate stiffness measurements in the frozen–thawed fibrotic/fibrosis-induced hPCLS as an advantageous biomarker and surrogate for targeting IPF drug development. This premise is supported by our results using the anti-fibrotic compound, Nintedanib, as co-treatment with this compound inhibited FC-induced stiffening (Figure 5c,d). 

We acknowledge six weaknesses in our current study. First, we are currently limited to a small number of fibrotic lung donors with different fibrotic subtypes. Second, we have not pre-sorted fibrotic hPCLS based on honeycombed vs. normal lung regions, which may have contributed to sample-to-sample variability in stiffness. Third, our current measurements are based on submerged, static hPCLS cultures. Fourth, we have limited toxicity testing to LPS as a tool to demonstrate test system responsiveness. Fifth, the cryopreservation time for the fibrotic hPCLS was limited to two weeks. Sixth, our data do not include microphotographs of FC-treated and fibrotic donor hPCLS. We seek to overcome these limitations in future studies by (1) enlarging our tissue repository, (2) pre-sorting prior to cryopreservation based on microscale morphology and/or stiffness, and (3) incorporating realistic hPCLS cues, including the air–liquid interface [53], stretch [26], ideally, applied in combination, and (4) expand the portfolio of toxicants tested to demonstrate the diverse response profiles that we believe hPCLS can deliver within both FC-treated frozen–thawed healthy hPCLS or frozen–thawed IPF tissues. (5) We anticipate that longer storage times are plausible because in previous studies using cryopreserved non-IPF hPCLS, we observed that storage durations as long as 34 weeks did not affect hPCLS viability post-thawing [46]. Moreover, these cryopreserved non-IPF hPCLS retain robust pro-fibrotic potential. (6) Initial indications suggest the FC treatment does not elicit overt histological changes (i.e., septal wall thickening) in the timeframe of treatment used. Additionally, hPCLS from fibrotic donors tend to have highly variable phenotypic expression of fibrotic markers that could not be presented in an effective manner for comparison with other datasets. Further refinement of fibrotic hPCLS evaluations may elucidate strategies that may allow histological discrimination between never-frozen and frozen–thawed hPCLS that are FC-treated or obtained from fibrotic donors. However, photomicrographs taken from a normal donor (not included in the fibrosis study) are included to demonstrate the similarity of never-frozen and frozen–thawed hPCLS cultures at day 1 and day 15 (Supplementary Figure S1).

## 5. Conclusions

Viable preparation of thin fibrotic and fibrosis-induced hPCLS has been at the forefront of pre-clinical IPF studies. However, widespread adoption has been hampered by limited tissue viability, as these samples typically lose viability one week after harvesting. Here, we have demonstrated that cryopreservation is a feasible solution to the problem of limited hPCLS viability. In addition, we have confirmed that realistic progressive fibrosis can be induced in frozen–thawed non-fibrotic hPCLS by treatment with an FC, and that the fibrotic induction can be quantified by functional measurements of hPCLS stiffness. The demonstration of these critical test system characteristics has positioned fresh and cryopreserved hPCLS derived from multiple donors to serve as models for evaluating anti-fibrotic therapeutic efficacy (or potential adverse effects), as well as to study the pro-fibrotic potential of environmentally prevalent materials that affect healthy lungs or create further risk to the IPF population. More generally, our data support the adoption of frozen–thawed hPCLS as an advantageous model system for acute toxic testing.

## Figures and Tables

**Table 1:** Donor Demographic Information.

Donor Number	Age	Race	Height (cm)	Weight (kg)	Sex	Lung Health Status
N1	66	Hispanic/Latino	157	61.7	M	Normal
N2	59	Hispanic/Latino	163	88.6	M	Normal
N3	60	White	168	83.7	M	Normal
N4	68	White	180	101.3	M	Normal
N5	69	Caucasian	175	79	M	Normal
D1	65	Hispanic	NA	NA	F	IPF/ILD
D2	61	Black	NA	NA	F	ILD/PF/PH
D3	31	Black	NA	NA	F	Fibrotic
D4	67	Caucasian	NA	NA	M	IPF/ILD
D5	54	Caucasian	NA	NA	F	IPF/ILD

## Data Availability

The data presented in this study are available on request from the corresponding authors.

## Supplementary Materials

The following supporting information can be downloaded at: https://www.mdpi.com/article/10.3390/toxics12090637/s1, Figure S1: Photomicrographs demonstrate the similarity of Never-frozen and Frozen-thawed hPCLS; Figure S2: Freezing-thawing the fibrotic hPCLS does not affect viability or protein content—Donor to donor breakdown; Figure S3: Freezing-thawing the fibrotic hPCLS largely preserves pro-fibrotic cytokine secretion—Donor-to-donor breakdown; Figure S4: Freezing-thawing the fibrotic hPCLS largely preserves pro-inflammatory cytokine secretion—Donor-to-donor breakdown; Figure S5: FC treatment of the frozen-thawed healthy hPCLS induces pro-fibrotic and pro-inflammatory secretion—Donor-to-donor breakdown; Figure S6:Nintedanib reduces FC-induced pro-fibrotic secretion in the frozen-thawed healthy hPCLS—Donor-to-donor breakdown.
