# Supplementary material for: The Fibrotic Phenotype of Human Precision-Cut Lung Slices Is Maintained after Cryopreservation
Source: Toxics. 2024 Aug 30;12(9):637. doi: 10.3390/toxics12090637 (PMC11436228; doi:10.3390/toxics12090637)
Supplement: Supplementary file 1 [file toxics-12-00637-s001.zip › Supplementary Figure Legends.pdf]

Supplementary Figure Legends

Supplementary Figure 1: Photomicrographs demonstrate the similarity of Never-frozen and Frozen-thawed hPCLS. Microphotographs from Never-frozen (top row) and ~8 months stored, Frozen-thawed (bottom row) hPCLS from donor N6. Tissues were cultured for 1 day (D1, left column) or 15 days (D15, right column) prior to fixation and staining. Few or no overt differences are noted between Never-frozen and Frozen-thawed, with tissues depicting excellent health. *H&E, 20X objective*

Supplementary Figure 2: Freezing-thawing the fibrotic hPCLS does not affect viability or protein content – Donor to donor breakdown. (a) WST-8 assay shows no significant difference between never-frozen and frozen-thawed groups across each of the 5 donors for up to 14 days in culture. Data are reported as the absorbance of WST-8 formazan at 450 nm, a value that is proportional to the number of viable cells in the medium. (b) BCA assay revealed no differences in general in protein concentration between the never-frozen and frozen-thawed groups for up to 14 days. Each bar is an average and standard deviation over n=6 hPCLS per donor for Frozen-thawed group and n=3 for Never frozen group. One-way ANOVA Sidak's test *:p<0.05, **: p<0.01, ****:p<0.0001. Donor information is provided in table 1.

Supplementary Figure 3: Freezing-thawing the fibrotic hPCLS largely preserves pro-fibrotic cytokine secretion – Donor-to-donor breakdown. (a-d) MAGPIX® analysis of cytokine production revealed that with limited exceptions, each of the pro-fibrotic cytokines were secreted comparably between the never-frozen and frozen-thawed fibrotic hPCLS at both D7 and D14. For several donors and cytokines, an increase in pro-fibrotic secretion was observed between 7 and 14 days in both the never-frozen and the frozen-thawed groups. Each bar represents mean and standard error of mean for each donor, with n=6 hPCLS per donor for Frozen-thawed group and n=3 for Never frozen group. *: p<0.05, **: p<0.01, ***:p<0.001. Donor information is provided in table 1.

Supplementary Figure 4: Freezing-thawing the fibrotic hPCLS largely preserves pro-inflammatory cytokine secretion – Donor-to-donor breakdown. MAGPIX® analysis of endogenous cytokine production revealed that compared to the never-frozen hPCLS, the frozen-thawed fibrotic hPCLS secreted similar levels of TNF-α (a) but significantly less IL-6 (b) at D7. However, these differences were absent at D14. (c-d) With subsequent LPS treatment, the average fold change (relative to pre-treatment) was comparable between the never-frozen and frozen-thawed fibrotic hPCLS at both D7 and D14. Each bar represents mean and standard deviation for each donor, with n=6 hPCLS per donor for Frozen-thawed group and n=3 for Never frozen group. One-way ANOVA, Sidak's test *: p<0.05, **: p<0.01, ***:p<0.001, ****:p<0.0001.

Supplementary Figure 5: FC treatment of the frozen-thawed healthy hPCLS induces pro-fibrotic and pro-inflammatory secretion – Donor-to-donor breakdown. (a-f) MAGPIX® analysis of cytokine production revealed an increase of the secreted cytokines after 2 and 4 days of treatment. For fibronectin, MMP-7 and MMP-3 the difference of increase between 2 and 4 days is significant. Each bar represents mean and standard deviation for each donor, with n=6 hPCLS per donor. Student, t-test : **: p<0.01,***: p<0.011 ****:p<0.001.

Supplementary Figure 6: Nintedanib reduces FC-induced pro-fibrotic secretion in the frozen-thawed healthy hPCLS – Donor-to-donor breakdown. MAGPIX® analysis of cytokine production

revealed that Nintedanib reduces FC-cocktail induced pro-fibrotic secretion with significant reduction in the cases of fibronectin (D4), pro-collagen Ia1 (D4), MMP-3 (D2 and D4), IL-6 (D4). We report fold change relative to donor-matched CC treated hPCLS. Each bar represents mean and standard error of mean for each normal donor, with n=6 hPCLS per donor. One-way ANOVA, Sidak's test, *:p<0.05, **: p<0.01,***: <0.011 ****:p<0.001.
