# Supplementary figures and images for: The Fibrotic Phenotype of Human Precision-Cut Lung Slices Is Maintained after Cryopreservation
Source: Toxics. 2024 Aug 30;12(9):637. doi: 10.3390/toxics12090637 (PMC11436228; doi:10.3390/toxics12090637)

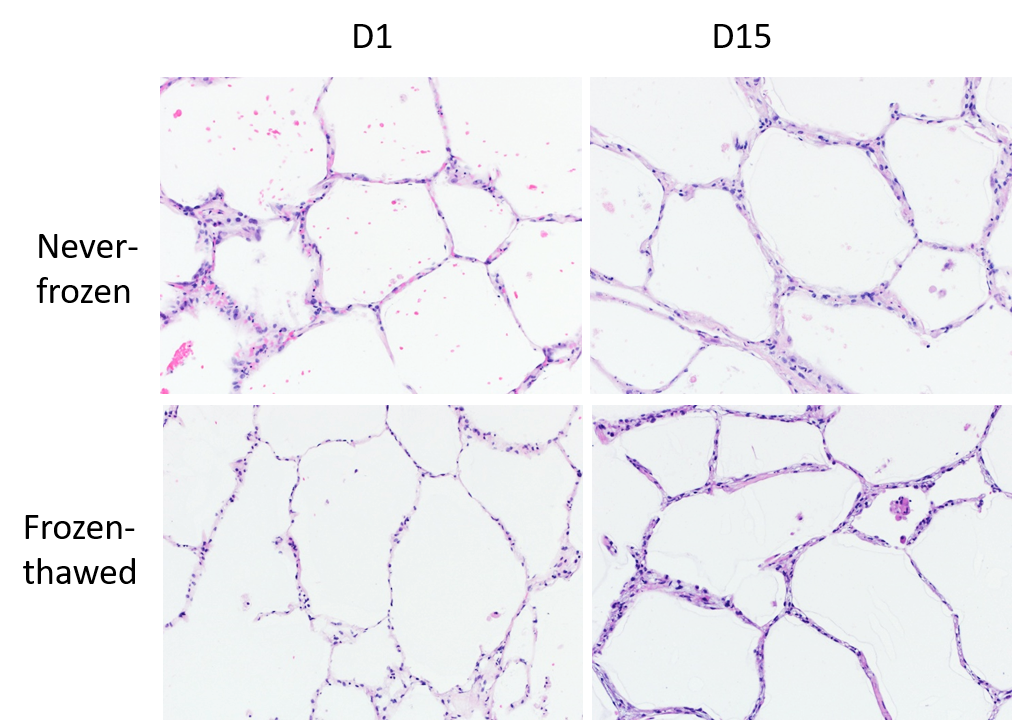

Supplement: Supplementary file 1 [file toxics-12-00637-s001.zip › Supplementary Figure 1-8-30.png]

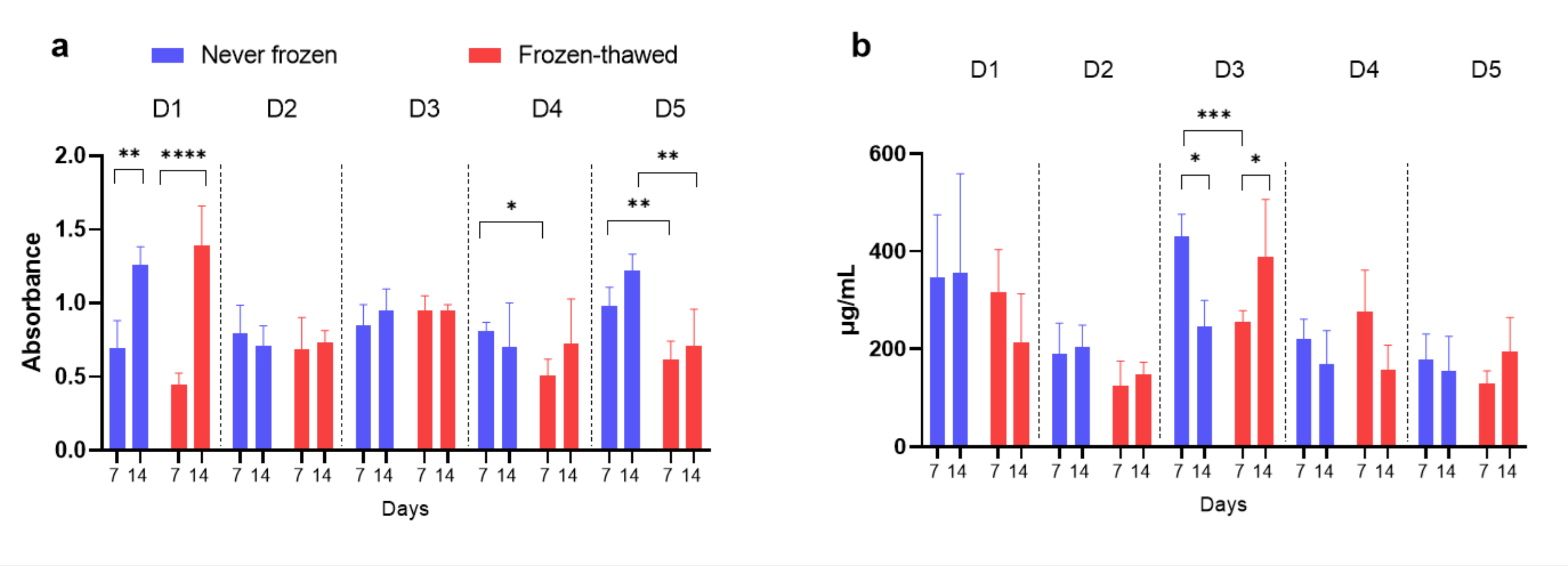

Supplement: Supplementary file 1 [file toxics-12-00637-s001.zip › Supplementary Figure 2-8-30.png]

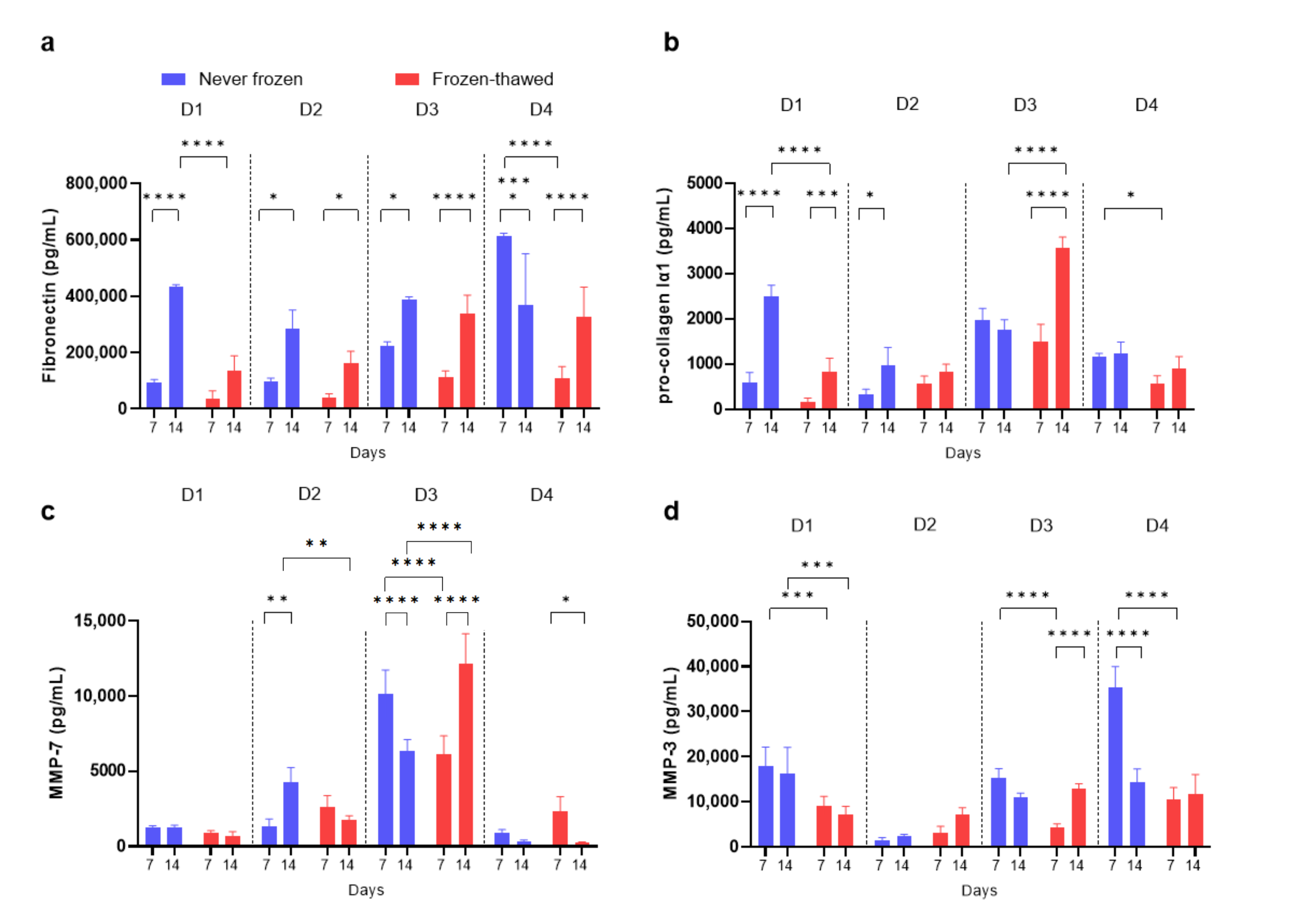

Supplement: Supplementary file 1 [file toxics-12-00637-s001.zip › Supplementary Figure 3-8-30.png]

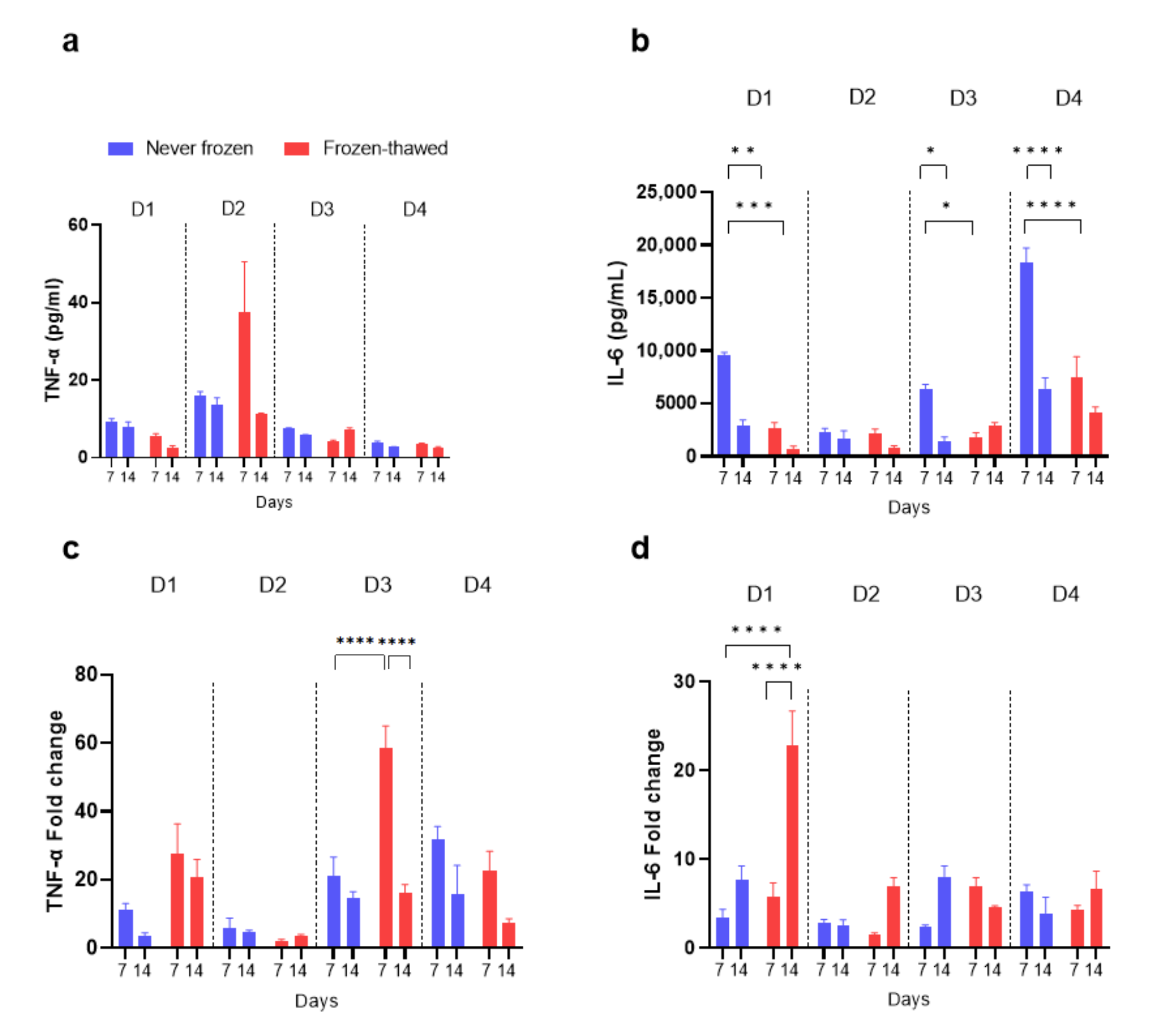

Supplement: Supplementary file 1 [file toxics-12-00637-s001.zip › Supplementary Figure 4-8-30.png]

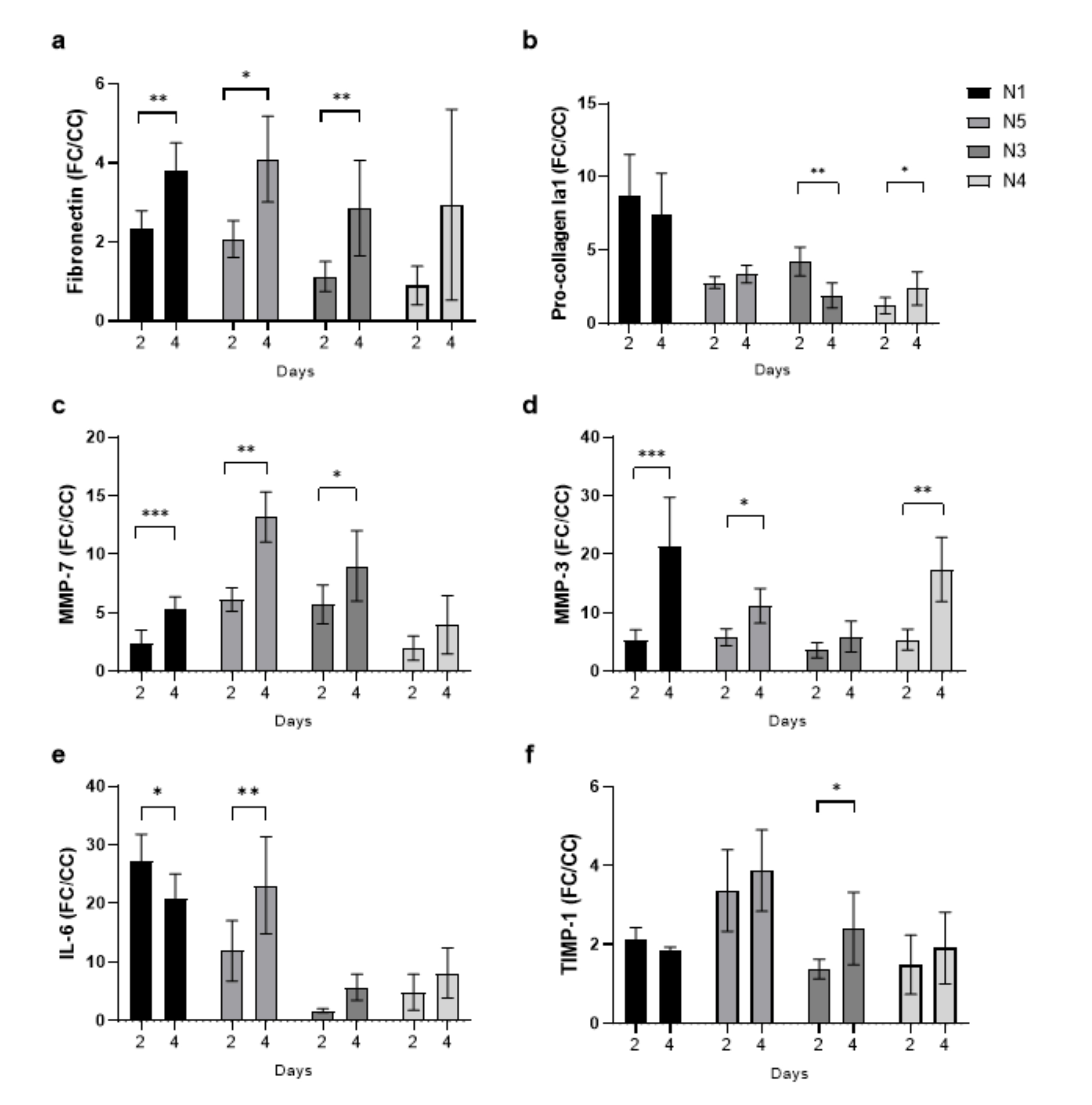

Supplement: Supplementary file 1 [file toxics-12-00637-s001.zip › Supplementary Figure 5-8-30.png]

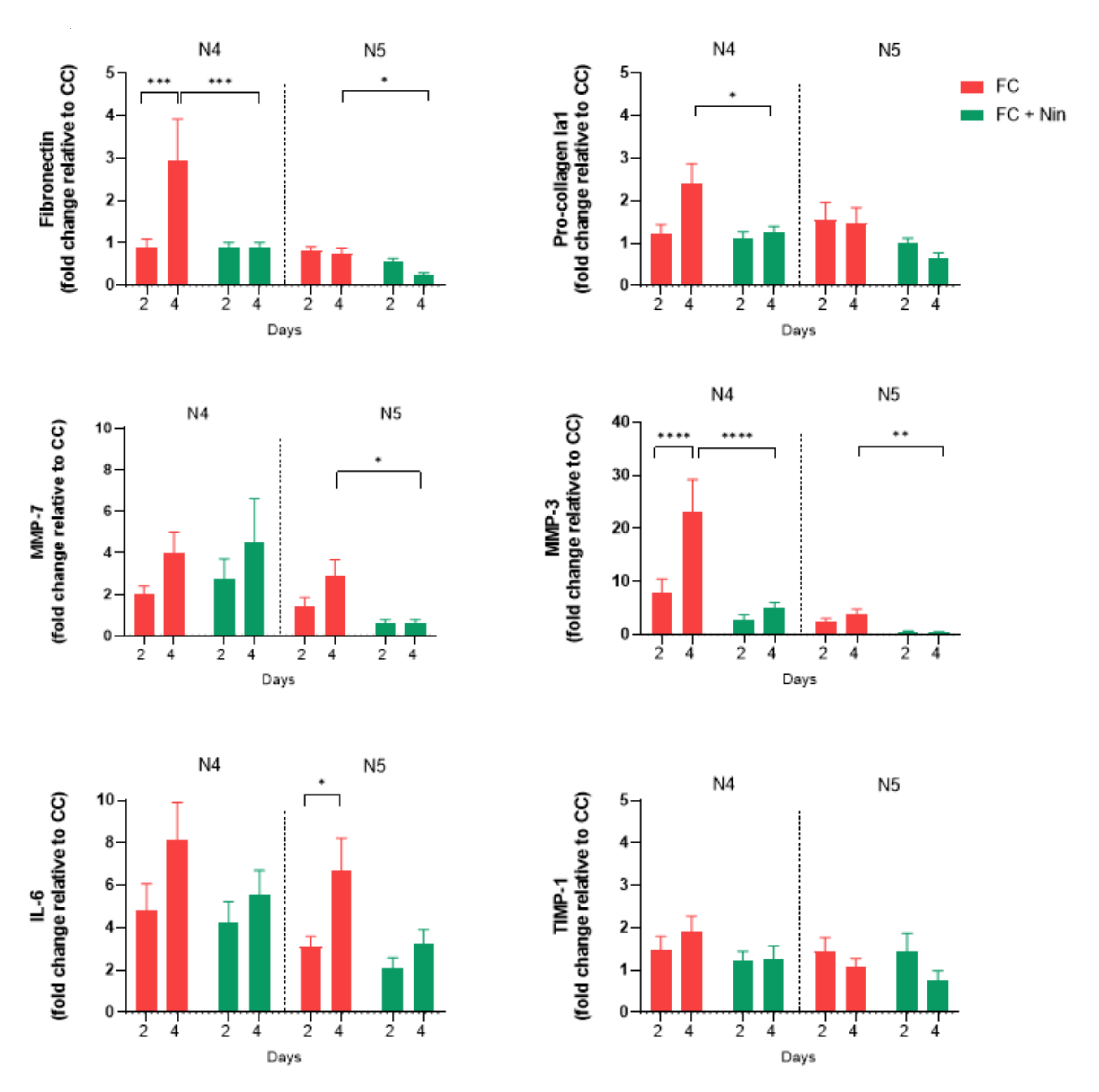

Supplement: Supplementary file 1 [file toxics-12-00637-s001.zip › Supplementary Figure 6-8-30.png]
